# Detailed causality between the valvular calcification and recurrent atrial fibrillation after catheter ablation

**DOI:** 10.1002/clc.24227

**Published:** 2024-02-12

**Authors:** Naoya Kataoka, Teruhiko Imamura

**Affiliations:** ^1^ Second Department of Internal Medicine University of Toyama Toyama Japan

To the Editor

1

The relationship between cardiac calcification and cardiovascular events is well‐established, although the clinical impact of valvular calcification on arrhythmia incidence remains unclear. In their study, Liu et al. demonstrated that valvular calcification independently increased the risk of recurrent persistent atrial fibrillation following radiofrequency catheter ablation.^1^ However, several points warrant further exploration.

While the correlation between mitral valve calcification and left atrial remodeling, elevated left atrial pressure, and atrial fibrillation development seems plausible, it remains uncertain why other forms of valvular calcification were similarly linked to atrial fibrillation recurrence. Additionally, the authors could shed light on why calcification of the pulmonary valve did not exhibit an association with recurrent atrial fibrillation.^1^

The exclusion of individuals with rheumatic heart disease raises methodological questions.^1^ Further elaboration on the methodology for their exclusion would be beneficial, particularly considering the challenges in distinguishing rheumatic valvular disease from other forms of valvular pathologies. Notably, rheumatic fever can give rise not only to mitral stenosis but also to mitral regurgitation.^2^

In the study, the authors dichotomized the presence of valvular calcification.^1^ While acknowledging that computed tomography stands as the gold standard for quantifying cardiac calcification,^3^ it would be advantageous if the authors quantified the degree of valvular calcification, treating them as continuous variables. Otherwise, assuming approximately 90% of the participants had no valvular calcification could raise concerns regarding the accuracy of their conclusions.^1^

Addressing the practical implications of their findings is pivotal.^1^ It would be valuable for the authors to propose potential therapeutic or prophylactic strategies for individuals with valvular calcification. For instance, should additional linear ablations be considered alongside conventional pulmonary vein isolation?

## CONFLICT OF INTEREST STATEMENT

The authors declare no conflicts of interest.
